# Complementary/Alternative Medicine in Cardiovascular Diseases 2013

**DOI:** 10.1155/2013/538346

**Published:** 2013-12-22

**Authors:** Ke-ji Chen, Ka Kit Hui, Myeong Soo Lee, Hao Xu

**Affiliations:** ^1^China Heart Institute of Chinese Medicine, Xiyuan Hospital, China Academy of Chinese Medical Sciences, Beijing 100091, China; ^2^Center for East-West Medicine, University of California, Los Angeles, Santa Monica, CA 90404, USA; ^3^Medical Research Division, Korea Institute of Oriental Medicine, Daejeon 305-811, Republic of Korea

Cardiovascular diseases (CVDs) are the leading cause of death worldwide, and the mortality is likely to further accelerate in many developing countries. In 2008, almost one in three deaths all over the world was attributed to CVDs. It is also estimated that by 2030 over 23 million deaths from CVDs will occur each year. Despite advances in modern management of CVDs, either revascularization or medical therapy, complications related to these procedures and recurrent acute cardiovascular events still afflict patients. Meanwhile, patients with CVDs often suffer from unfavorable quality of life. In recent decades, the potential benefit of complementary/alternative medicine (CAM) therapy in improving CVDs prognosis has drawn more and more attention, and the use of CAM by physicians and patients has also increased markedly. However, the evidence of CAM for CVDs patients and the research on mechanism of actions are still insufficient. Last year, the published special issue named “*The potential benefit of complementary/alternative medicine in cardiovascular diseases*” got a great success, which facilitates the compilation of this special issue 2013. We believe that such a series can have a long-term impact, and in time gather a community around it in much the same way a successful annual conference does.

In this issue, original research papers and reviews from different parts of the world including China, Republic of Korea, USA, Germany, and Malaysia are presented. These papers are focused on the mechanism of action and the clinical application of CAM in treating CVDs. In the clinical trials, the benefits of Chinese medicine and some other CAM therapies for CVDs patients were demonstrated. A multicenter prospective cohort study showed that heart failure, age ≥65 years old, and myocardial infarction were associated with an increase in one-year follow-up incidence of major adverse cardiac events (MACEs) in hospitalized coronary heart disease patients, and integrative medicine showed a tendency for reducing the incidence of MACEs. The similar benefit of integrative medicine therapy for patients with acute coronary syndrome after PCI was showed in a multicenter randomized controlled trial (RCT), 5C trial. Another RCT compared the effectiveness of Qi-shen-yi-qi dripping pills (QSYQ) with that of aspirin in the secondary prevention of myocardial infarction. This trial did not show significant difference of primary and secondary outcomes between aspirin and QSYQ, which suggest QSYQ might be an alternative medication for patients' intolerance of aspirin in patients who have had an MI. Specifically, a new potential biomarker of “toxin syndrome” in coronary heart disease patients was proposed in a paper containing two clinical trials. The authors concluded that the new biomarker “inter-alpha-trypsin inhibitor heavy chain H4” might have a potential role in early identifying high-risk coronary heart disease patients in stable period. Most of the experiment researches in this issue were focused on Chinese medicine and Korean medicine. Two researches explored the role and mechanism of Qiliqiangxin capsule (QL), an oral Chinese proprietary medicine, for arrhythmia and heart failure, respectively. A pharmacological research showed that blocking androgen receptor could abolish the ability of Panax ginseng to protect the heart from myocardial ischemia reperfusion injury. The benefit of Doinseunggitang, a Korean traditional prescription, on the treatment and prevention of diabetic vascular complications was also described in a study. A systematic review suggested that oral Panax notoginseng preparation could relieve angina pectoris related symptoms. Meanwhile, the potential benefit of Qiju Dihuang Wan, a Chinese herbal prescription, on the treatment of essential hypertension was also reviewed. Additionally, yoga was recommended as an effective intervention for reducing blood pressure in a systematic review. 

Due to indefinite mechanism of actions and lacking of high quality evidence, traditional Chinese medicine and other traditional medicine worldwide are considered as CAM in Western countries. In this case, original researches on mechanism of actions play an important role in the modernization and internationalization of CAM and should be further strengthened in future researches. In the hierarchy of evidence-based medicine, high quality RCT is still considered as the golden standard for evaluating interventional treatment. Currently, multicenter RCTs and a prospective cohort study have been conducted to evaluate the benefit of CAM for CVDs, and most of these researches showed positive findings. Nevertheless, the effectiveness of CAM on the treatment of CVDs in the real world is still unclear which limits the application of CAM. The reason is that efficacy of intervention in RCT is not equal to its effectiveness in the real world. In the future, in addition to high quality RCTs, more evidence from real world researches is warranted to support the use of CAM including traditional Chinese medicine for CVDs patients.

*Ke-ji Chen* *Ke-ji Chen* *Ka Kit Hui* *Ka Kit Hui* *Myeong Soo Lee* *Myeong Soo Lee* *Hao Xu* *Hao Xu*

